# Snip, Support, and Shared Stories: Exploring Reddit Users' Experiences With Vasectomy

**DOI:** 10.7759/cureus.71374

**Published:** 2024-10-13

**Authors:** Max D Sandler, Jordan C Best, Mary K Samplaski, Armin Ghomeshi, Thomas A Masterson

**Affiliations:** 1 Desai Sethi Urology Institute, University of Miami Miller School of Medicine, Miami, USA; 2 Institute of Urology, University of Southern California Keck School of Medicine, Los Angeles, USA; 3 Department of Urology, Florida International University Herbert Wertheim College of Medicine, Miami, USA

**Keywords:** erectile dysfunction, patient counseling, qualitative research, sexual dysfunction, vasectomy

## Abstract

Introduction

Vasectomy is a common procedure for male sterilization. During pre-procedural counseling, men and their partners are often concerned with postoperative pain or sexual dysfunction. Research examining internet forums to explore these concerns is limited. In this study, we selected Reddit, a popular and anonymous online platform for user discussion, to qualitatively investigate patient experiences and questions surrounding vasectomy so healthcare providers may better understand patient concerns.

Methods

Threads, composed of a primary post to the website and associated comments, are organized into topic-focused forums known as subreddits. We collected threads from the Reddit group "Vasectomy" over a 12-month period. Terms searched included "pain," "volume," "erect," and "ED." Threads not focused on these terms were excluded. Two investigators trained in qualitative research used a modified grounded theory to independently code threads using key phrases. Similar phrases were grouped to form themes, which were refined into concepts.

Results

Threads discussed pain, ejaculate volume, and erectile dysfunction (ED) after vasectomy. An analysis of 87 threads with 1,052 responses revealed three themes: men on Reddit seek validation, recount their vasectomy experiences, and share anxieties. Concepts suggest men utilize the community to cope with these anxieties and that a discrepancy exists between expectations set by physicians and patients' actual postoperative experiences.

Conclusions

This study provides valuable clues about patients' perspectives on vasectomy and the information they seek or share online. Discrepancies exist between patient expectations and guidance provided by urologists, suggesting a need for more tailored preoperative counseling. By actively acknowledging concerns about vasectomy, healthcare providers may potentially be able to better understand and cater to patient needs.

## Introduction

Vasectomy is a common surgical procedure for permanent male contraception. A total of 500,000-600,000 vasectomies are performed in the United States [1]. The American Urological Association guidelines report that vasectomies are relatively low risk; about 1%-2% of men experience chronic post-vasectomy pain and an associated decreased quality of life [2]. Advancements in procedural techniques have decreased the incidence of postoperative pain and complications such as hematomas, infections, and sperm granulomas to approximately 1%-2% of cases [3].

During pre-procedural counseling, commonly reported concerns of men and their partners revolve around postoperative pain or sexual dysfunction, such as changes in ejaculation characteristics or erectile dysfunction (ED) [4-6]. Published qualitative studies have employed surveys and interviews to investigate patient perspectives on vasectomy, yet there remains a paucity of research examining internet discussion groups to better understand men's concerns and experiences with the procedure [7-10]. Analyzing online forums is an opportunity to gain insight into the questions patients ask others who have undergone the procedure, as they may be uncomfortable asking their provider.

Given the recent restrictions on women's reproductive rights, public interest in vasectomy as a form of contraception has risen significantly [11]. Studies show significant increases in men under 30 seeking vasectomy, as well as the total number of vasectomy consultations and procedures since the overturning of Roe v. Wade, the federally protected right to abortion [11]. With approximately 52 million daily active users, Reddit is a popular social media platform for user discussion, especially among people of reproductive age, and has been used in other studies to explore patient experiences [12-15]. In this study, Reddit was selected as a vehicle to investigate patient experiences, emotions, and questions surrounding vasectomy. This study attempts to aid physicians in identifying patient information needs so that they may provide evidence-based guidance, rather than anecdotal sources that patients often rely on [10,16]. The objective of this research is to analyze data posted to an anonymous online forum for thematic content using an established qualitative method. In doing so, our goal is to explore men's experiences and expectations with vasectomy in order for healthcare providers to better understand patient concerns.

## Materials and methods

Reddit, an extensive network of highly active and user-moderated forums, was selected as the source of vasectomy discussion because this platform is used by millions to inquire about a wide variety of topics. Reddit threads, each composed of a primary post and comments, are organized into topic-focused forums known as subreddits. Specifically, we analyzed threads from the subreddit "r/Vasectomy," where its 15,000 members discuss questions regarding vasectomies and experiences with the procedure. After filtering to show posts within the last year, the researchers browsed r/Vasectomy to gain a general understanding of frequently discussed and popular topics.

To identify relevant posts, the researchers selected search terms that reflected common discussions on r/Vasectomy and the language patients typically use during appointments to express concerns about vasectomy. We collected specific threads to analyze by searching the terms "pain," "volume," "erect," and "ED" within r/Vasectomy. Any thread where the primary post was not focused on these terms was excluded. Vasectomy-related threads were filtered and collected over a 12-month period, from February 6, 2023, to February 24, 2024. We collected threads for each term until we reached saturation, meaning no new ideas emerged and the content of the threads became repetitive.

Titles, descriptions, the number of comments, and the date of each post were inputted into an online spreadsheet software. Two investigators, with training in qualitative research, each reviewed and independently coded relevant threads by using key phrases to identify the main ideas discussed in each post and its associated comments. Thematic analysis was then conducted by grouping together similarly coded threads. Themes emerged through the subsequent review of these groups based on qualitative content analysis, specifically a modified grounded theory. Grounded theory, a method of inductive reasoning, was used in this study to determine a research focus and create a basis for subsequent studies [17,18]. The investigators refined and categorized all themes into general concepts. Through discussion, both researchers came to a consensus on these themes and the resultant concepts. This study was exempt from institutional review board approval.

## Results

We identified 87 distinct threads, with 1,052 responses. Of these threads, four were found by searching "ED," four by "erection," 58 by "pain," and 21 by "volume." As Reddit is an anonymous and voluntary forum, there was no demographic information available. The study population was composed of members of the subreddit "r/Vasectomy." We identified three primary themes: preoperative concerns, seeking reassurance, and venting frustrations or recounting experiences to inform others (Table 1).

**Table 1:** Themes (top) generated from the analysis of threads on the subreddit r/Vasectomy, with excerpts of posts, which informed the creation of these themes.

Preoperative Anxieties	Seeking Reassurance	Sharing Experiences
"I'm 26 and was planning on getting the snip this year; however, I just heard a couple of other guys that got them around my age…are now apparently experiencing ED about 5-10 years later"	"Am I getting in my own head because it happened twice and now I'm afraid during sex but when I'm alone I'm comfortable and able to perform or have other ppl experienced ED three or so months post-op?"	"Maintaining an erection is a lot harder; if I don't orgasm when I remove my hand from myself, I'll go completely soft within 10-20 seconds (meaning even the period of 'losing an erection' is far more rapid than before…)"
"Am I right in assuming the brochure information of what to expect (regarding pain) is very optimistic but in truth is not realistic?"	"Volume is definitely less…my wife has also noticed. I know sperm doesn't count for much of the volume, but mine is noticeably less. Has anyone seen improvements with this as time goes on?"	"I personally found comfort in finding other people who had similar pain at similar times. I started a pain log…figured I would also post here if it could help someone else see the light at the end of the tunnel"
"I have one concern though; will the size and volume of my (ejaculate) be effected?"	"I wake up at night sometimes from burning pain…. Any tips and support are welcome. Just needed to tell someone about the struggle"	"If volume and orgasms return to normal or even improve and get closer to where they used to be, then that will be a bonus"

Ten posts with 222 associated comments surrounded preoperative concerns, which is the first theme. Men inquired about whether they should expect significant pain during or after the procedure, as well as if semen volume or erectile function is affected by vasectomy. In terms of pain, many users in the comments of posts acknowledged that there is no single, universal response to vasectomy, and pain can vary in both intensity and duration. Many provided anecdotal tips to reduce discomfort as well, such as over-the-counter analgesics, ice, and avoiding strenuous activity. Regarding sexual dysfunction posts, commenters primarily reported no changes in these domains and offered alternate explanations for ED after vasectomy. Some commenters provided references to supporting literature, and others recommended discussing it with a physician.

The second theme focused on postoperative posters to confirm that the pain, reduced ejaculate volume, or ED they were experiencing was shared by others. Fifty-four distinct posts sought reassurance, and 529 comments contained similar requests or served to reassure the poster. Posts typically included variations of the phrase "anybody else?" The vast majority of pain-related posts were of this theme; men who experienced pain, anywhere from the day of the operation to years later, were posting with the goal of soliciting solidarity from others in similar situations. In these posts, more than 10 commenters expressed their mistrust of healthcare providers and implied that they were not adequately counseled on the range of intra- or postoperative pain that they experienced.

In response to posts seeking reassurance about perceived semen volume changes, several comments asserted that sperm makes up too small a percentage to affect ejaculate volume in any observable way, and changes were likely related to diet and hydration status. A few commenters cited Nikkanen's 1979 study explaining that while viscosity may change, volume decreases are insignificant after vasectomy [19]. Other posts sought reassurance about the erectile function being impaired after vasectomy. Users suggested that this may be a natural part of the recovery process or that psychological and overall health factors may be to blame. Two commenters endorsing ED expressed distrust in their urologist for suggesting erections are unrelated to vasectomy.

The third and final theme captured a blend of positive and negative experiences shared by users regarding vasectomy. This theme spanned 23 threads and included 301 comments. Sixteen (69.6%) posts featured a mix of positive and negative perspectives shared by patients who had undergone vasectomy; those with positive experiences expressed their optimism that their stories will motivate others who are ambivalent or learning more about vasectomy. Seven (30.4%) distinct posts exclusively detailed the author's negative experience with vasectomy and were labeled as "venting" posts, serving as an emotional release. Negative posts also included users surprised or upset by the mismatch between what their urologist told them to expect and the postoperative changes they experienced, as well as instances of physicians minimizing their concerns. Posts chronicling unexpected, lasting pain were met with some commenters noting less pain compared to the original poster. These commenters indicated their desire to alleviate concerns for those viewing the negative post and may be worried about their own recovery. However, many others empathized with similar, painful experiences and reported wariness of expectations set by urologists: "this procedure, and recovery, sure isn't as quick and easy as they make it out to be, is it?"

Posters venting their disappointment with reduced volume were often met with reassurance or dismissal, while comments responding to complaints of ED primarily focused on providing explanations, such as postoperative pain or psychological stress. Positive posts were sometimes tagged as public service announcements (PSA) for the education of other users. One user was pleased with the lack of pain he experienced and posted the following: "Seriously! If you're worried about pain, don't be. If it was a procedure I had to get done every year, it would still be worth it." In four unique positive posts, users mention that their volume has not been affected; either these posters were worried about volume changes before their own vasectomy or they recognize that other men may be.

We developed general concepts based on these three themes (Table 2). The first serves to reinforce that men utilize the community as a coping mechanism; many patients experience inherent anxieties regarding vasectomy and search for validation regarding their concerns about this procedure. The second concept revolves around information discrepancy between expectations set by healthcare professionals and the actual postoperative experiences shared by the patients.

**Table 2:** Concepts (top) that formed upon the analysis of themes, with depictive excerpts from relevant threads on the r/Vasectomy subreddit.

Coping and Community	Expectation Versus Reality
"The quality of my erection and orgasm was poor compared to before, but I know I'm still healing, and I'm not too worried about it yet"	"My ejaculate volume is lower than it used to be…. I was always told that the operation wouldn't affect these things at all"
"Same here and my velocity/volume never returned, after ~9 years and counting"	"This was unexpected as nearly everything I was told and read indicated there would be no perceptible change"
"Don't discount too much that it is not just in your head…you may experience some loss of confidence or added stress that could affect your performance"	"(The urologist) said…this was not likely to be causing the ED/ejaculation problems. I can't imagine how the two are not related (vasectomy & ED)"
"I'm just looking for some reassurance here…; please oh please, someone let me know I'm not alone in this!"	"2 different urologists…said it was all in my head…. I went ahead with the reversal, and I'm having strong erections…. Vasectomy isn't as risk free as they claim it is"
"I wish you luck…; the key is to not get anxious about it and take it as it comes"	"The doctors lie about the rarity of the complications. They definitely do"
"Has anyone had any of this happen? It's not incredibly painful, but it's enough to stress me out"	"The internets need to be updated on vasectomy recovery"
"Don't forget to share your experience with us!"	"Can takes months. Docs should give better info about the wide range of recovery time"

## Discussion

The internet is a commonly used resource for health information among people of reproductive age [20]. Several recent studies have used Reddit to qualitatively analyze urological conditions and found that many patients visit the website seeking answers and peer support [13-15,21]. This popular, anonymous discussion board enables users to express their concerns freely, some of which they may otherwise be uncomfortable discussing with medical professionals in person. Through Reddit, we sought to gain insight into patient perspectives on vasectomy, pain, and its perceived associations with ED or ejaculate volume changes. The objective of this research is to understand the specific information patients seek on the internet related to vasectomy, enabling clinicians to provide more comprehensive and tailored counseling to patients.

Using individual and group interviews, prior work by Shih et al. found that both men and women reported a fear of lasting changes in sexual function as a result of vasectomy [9]. In a 1991 survey conducted by Thompson et al., couples opting for female sterilization over vasectomy cited concerns over adverse effects on their sex life post-vasectomy [22]. In this study, we found that pre- and postoperative individuals visited the subreddit "r/Vasectomy" looking for a sense of community and to be reassured by those navigating similar experiences. These findings are similar to Samplaski's 2018 study of 129 posts about vasectomy as observed through similar online discussion boards, which found that patients were primarily seeking support regarding sexual function changes, as well as vasectomy-related pain [10].

Online peer support reduces social isolation and has been shown to be associated with improvements in depression, anxiety, quality of life, and self-efficacy [23,24]. However, user-organized online forums run the risk of exposure to misleading information or hurtful and invalidating posts. Healthcare providers must be aware that men are utilizing online peer support for peri-vasectomy information, which may or may not contain accurate medical knowledge or create further anxieties. Another finding from this study was that men share their vasectomy-related experiences for the benefit of others who may have similar questions. Pre-, peri-, and post-vasectomy men posting their experiences online can aid providers in gaining a comprehensive understanding of individual needs and challenges, as well as contribute to the development of patient-focused education [13].

Importantly, there appears to be an information gap between the patient's experience after vasectomy and the expectations urologists may create. Reddit users expressed frustration when the side effects they experienced did not align with what they had anticipated based on the counseling provided by their urologist. Prior research indicates that chronic scrotal pain after vasectomy is fairly common and affects one in seven men [25]. This contrasts with more recent guidelines published by the American Urological Association, which states that vasectomy-related pain severe enough to impact the quality of life occurs in 1%-2% of patients [26]. Evidence indicates that vasectomy does not lead to changes in semen volume or cause erectile dysfunction [27,28]. However, other research shows that ED is considered to be a significant psychological post-vasectomy problem [29]. These discrepancies and resultant distress in patients with post-vasectomy sexual dysfunction or longer-than-expected pain lead them to seek alternative sources of information, such as online forums.

Urologists and other healthcare workers must ensure that pre-procedural patients clearly understand the risk of pain associated with vasectomy. While the authors note evidence of small-study effects and heterogeneity of studies, a 2020 systematic review and meta-analysis found that the overall incidence of chronic pain presenting more than two weeks following no-scalpel vasectomy was 7% (95% CI: 4%-13%) [30]. Providers should also acknowledge perceptions of sexual dysfunction after vasectomy and carefully educate patients that numerous studies link vasectomy and erectile dysfunction to psychosocial factors rather than the physical effects of the procedure [5].

In Singh et al.'s 2014 article, diagrams of male anatomy were employed to teach 600 healthcare providers that erection, ejaculation, and orgasm are unaffected by vasectomy, with the only change being the absence of sperm in seminal fluid. Prospective vasectomy patients from cultures where discussing sexual function is taboo expressed relief when healthcare workers initiated these conversations, with some reporting the provided information to be just what they wanted to hear [6]. In a 2012 interview-based qualitative study, both men and women described ideal counseling on vasectomy as including specifics on pain expectations and sexual function changes [8]. Together with the findings of our study, this implies that providers should focus educational initiatives on post-vasectomy expectations during counseling and furnish patients with credible online sources delineating the circumstances warranting medical attention following the procedure.

The limitations of this study include the self-selection of participants on the "r/Vasectomy" subreddit. Reddit users must have access to and be comfortable enough with technology to post their intimate thoughts and feelings on Reddit, which may exclude those unfamiliar with the platform. Another factor of self-selection bias is that the men who had concerns or issues related to vasectomy may not accurately represent the broader vasectomy experience. Due to its anonymous nature, we were unable to confirm demographics, clinical history, and physical examination or ask clarifying questions. As these men are self-identifying by participating in discussions on vasectomy, it is impossible to verify whether their symptoms are exaggerated, underplayed, or otherwise unrelated to vasectomy. This study focused on posts containing specific search terms that aligned with frequent discussions on Reddit and how patients typically address these topics. However, the choice of these terms influenced the common themes identified, potentially leading to the omission of other relevant themes in Reddit vasectomy posts that were not reviewed. Another limitation is that we only collected one year's worth of threads from a single website; there exist other discussion boards on the internet with a vasectomy-focused discourse that we did not analyze, such as those examined by Samplaski [10]. To maximize reliability and minimize bias, two independent researchers used the established, validated method of grounded theory, as described by Charmaz, to collect and analyze data [18].

## Conclusions

Employing the qualitative analysis of a popular social media platform, we revealed that men seek online communities for reassurance, share preoperative anxieties, and discuss frustrations or experiences to educate others. These themes may suggest that men look for others to validate their concerns about the procedure and use online communities as a coping mechanism. Particularly significant was the existence of an information gap between expectations set by healthcare professionals and some patients' actual postoperative experiences, especially concerning sexual dysfunction and pain after vasectomy. This insight may encourage providers to tailor educational efforts to acknowledge and address these concerns. Despite limitations, insights from Reddit threads offer valuable clues about patients' inquiries and the information they encounter. Future investigations could employ structured, anonymous data collection from deidentified patients to deepen the understanding of vasectomy-related worries and questions. By actively listening to patient concerns and experiences surrounding vasectomy, healthcare providers may potentially be able to better cater to their needs.
